# An Automated Room Temperature Flip-Chip Mounting Process for Hybrid Printed Electronics

**DOI:** 10.3390/mi13040583

**Published:** 2022-04-08

**Authors:** Zehua Chen, Ulrich Gengenbach, Xinnan Liu, Alexander Scholz, Lukas Zimmermann, Jasmin Aghassi-Hagmann, Liane Koker

**Affiliations:** 1 Institute of Automation and Applied Informatics, Karlsruhe Institute of Technology, Hermann-von-Helmholtz-Platz 1, 76344 Eggenstein-Leopoldshafen, Germany; ulrich.gengenbach@kit.edu (U.G.); xinnan.liu@student.kit.edu (X.L.); liane.koker@kit.edu (L.K.); 2 Institute of Nanotechnology, Karlsruhe Institute of Technology, Hermann-von-Helmholtz-Platz 1, 76344 Eggenstein-Leopoldshafen, Germany; alexander.scholz2@kit.edu (A.S.); jasmin.aghassi@kit.edu (J.A.-H.); 3 Hahn-Schickard-Gesellschaft für angewandte Forschung e.V., Wilhelm-Schickard-Straße 10, 78052 Villingen-Schwenningen, Germany; lukas.zimmermann@hahn-schickard.de

**Keywords:** bump-less bonding, conductive adhesive interconnection, flip chip, standardized interconnection, hybrid printed systems, room temperature bonding, automated mounting process, dispensing process influences, inkjet printing, flexible hybrid electronics

## Abstract

Printing technology and mounting technology enable the novel field of hybrid printed electronics. To establish a hybrid printed system, one challenge is that the applied mounting process meets the requirements of functional inks and substrates. One of the most common requirements is low process temperature. Many functional inks and substrates cannot withstand the high temperatures required by traditional mounting processes. In this work, a standardized interconnection and an automated bump-less flip-chip mounting process using a room temperature curing conductive adhesive are realised. With the proposed process, the conductive adhesive selected for the standardized interconnection can be dispensed uniformly, despite its increase of viscosity already during pot time. Electrical and mechanical performance of the interconnection are characterized by four terminal resistance measurement and shear test. The herein proposed automated process allows for fabrication of hybrid printed devices in larger batch sizes than manual assembly processes used beforehand and thus, more comprehensive evaluation of device parameters. This is successfully demonstrated in a first application, a novel hybrid printed security device. The room temperature mounting process eliminates any potentially damaging thermal influence on the performance of the printed circuits that might result from other assembly techniques like soldering.

## 1. Introduction

Printed electronics are an emerging technology that offers formerly unknown possibilities for product design by using non-standard materials as inks or substrates [1,2,3,4]. Large-area low-cost fabrication on bendable or even stretchable substrates can be realized as well as on-demand customisation down to the quantity of one device. There is a variety of envisaged fields of applications reaching from health care over industrial implementation in human-machine interfaces up to environmental monitoring [5,6,7,8,9].

Printed electronics are fabricated by printing functional inks, frequently formulated from nanoparticles and solvents onto a substrate. In subsequent postprocessing steps (e.g., drying, sintering), the solvents are removed and the functional structure formed. To date, conductive traces, numerous sensors, passive components, transistors and batteries can be printed [10,11,12,13,14,15,16,17,18]. Still, printed devices alone frequently do not suffice to achieve full functionality. They have to be complemented by silicon devices mounted onto the substrate. With this hybrid setup, the most beneficial properties of functional printing and silicon-based electronics are combined. One common approach is called flexible hybrid electronics (FHE) [2,19]. For FHE, to date mostly standard electronic components are mounted on a flexible or bendable foil. Within the area of these rigid electronic components, however, the flexibility is limited. To retain the flexibility of the substrate, unpackaged silicon chips, so-called bare dies, can be applied that are thinned down to, e.g., 30 μm to also become flexible [20]. As many common foil materials do not stand elevated processing temperatures, low temperature mounting processes are a requirement for FHE. Besides FHE there are other types of hybrid printed systems whose constituent materials impose limits on silicon component mounting process temperatures. Examples are functional inks for active electronic devices [21,22,23].

For both types of hybrid printed systems, the printed device has to be connected to the silicon-based part of the system at low process temperatures down to room temperature. To meet this requirement, a scalable automated room temperature mounting process is developed in this work. In order to select the appropriate bonding technique, state of the art mounting technologies for unpackaged electronic components are investigated. For silicon bare dies, wire-bonding is well established [24]. Printing processes also show potential to replace the bond wires by printed conductive lines [25]. In these cases, however, the contact pads on the substrate are arranged around the circumference of die and thus claim additional surface area. Moreover, the functional surface of the die is mounted face up. Hence, both the die and the die-substrate connection (e.g., wirebond) have to be additionally protected by an encapsulation such as glob-top. Flip-chip technology has been developed to increase integration density and improve high-frequency properties of the die substrate connection [24]. Here, the die is mounted face down to the substrate. Thus, its functional surface is already protected from environmental influences. The two main process categories for flip-chip bonding are direct and indirect bonding [26]. Direct bonding is mainly used for bonding a chip to a wafer [27]. Indirect bonding requires an additional interconnection material between the two surfaces to be connected. In most cases, this additional interconnection material is a solder bump, which is already created on wafer level. An alternative is the formation of a pillar with a solder top. Finally, the deposited solder is reflowed to yield the desired spherical shape [28]. Soldering is a high temperature mounting process, since the interconnection is realized by melting the solder. Even low-temperature solders, e.g., consisting of tin (Sn) and bismuth (Bi), typically require processing temperatures above 130 °C [29]. Another class of bonding processes is based on either isotropic or anisotropic conductive adhesives as interconnection material [30]. Here, the solder bump is replaced by a dot of conductive adhesive deposited by a dispensing process (e.g., needle dispensing, jetting) on the pad. After mounting of the chip, the adhesive is cured either thermally at temperatures lower than solder melting temperatures down to room temperature or by illumination with ultraviolet light [25,31]. Hence, this class of interconnection materials and the associated deposition processes are gentle to both printed structures and substrates as there is no need for lithography and wet processes. Furthermore, by proper selection of the adhesive, the thermal and chemical strain during mounting and curing can be minimised. The drawbacks are the lower conductivity of the conductive adhesive compared to solder and corrosion issues. These issues are being addressed by improved conductive adhesive formulations, e.g., with silver (Ag) nanoparticles or carbon nanotubes to improve conductivity and oxygen scavengers to improve corrosion resistance [32]. Different from solder, applicability of conductive adhesive is not limited to the typical microelectronic metallisations such as aluminium (Al), copper (Cu), Ag, gold (Au). On the other hand, there is no self-alignment effect during mounting when using adhesive.

Due to the high requirements on low processing temperature for the intended applications, a room-temperature bump-less flip-chip mounting process based on isotropic conductive adhesive is chosen to realise the electrical and mechanical interconnections.

The first application is a novel security device, a physically unclonable function (PUF) [23]. The PUF core is based on a printed inverter array on ITO-coated glass and connected to a conventional PCB containing the read-out circuitry. Since the process is scalable, it can later be adapted in order to be applied to printed FHE or other hybrid printed systems.

## 2. Materials and Methods

In this section, the materials - conductive adhesive, substrates and the processes as well as related equipment for establishing the room temperature mounting process are outlined.

### 2.1. Materials

Isotropic conductive adhesive is used as interconnection material both with a PCB test vehicle to assess its mechanical and electrical interconnection properties and for mounting an ITO-coated glass containing printed structures onto an adapter PCB.

#### 2.1.1. Conductive Adhesive

The epoxy based silver-filled two-component room temperature curable adhesive Elecolit 325 from Panacol is selected as isotropic conductive adhesive. The target diameter of a conductive adhesive dot forming an interconnection between ITO-coated glass and adapter PCB is (1.0 ± 0.2) mm. Hence, Nordson EFD 7018333 red dispense needles with inner diameter of 250 μm and outer diameter of 520 μm are utilized for adhesive dispensing. The selected conductive adhesive allows low temperature bonding by curing 16 h at room temperature.

#### 2.1.2. Test Vehicle

**Figure 1:**
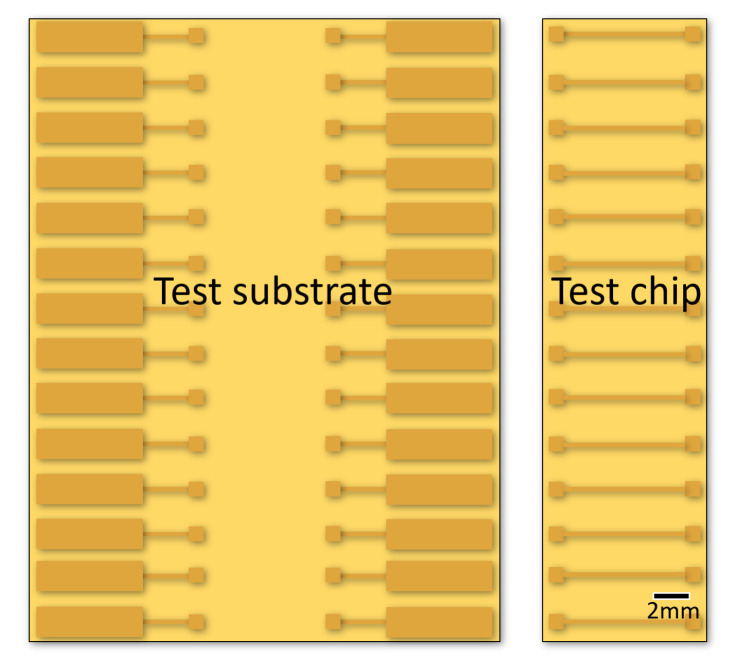
Schematic drawing of the test vehicle applied for the measurement of electrical and mechanical performance of conductive adhesive interconnection.

To investigate the electrical and mechanical performance of the conductive adhesive interconnection, a test vehicle is designed and fabricated from PCB material as shown in Figure 1. It consists of a test chip representing the ITO-coated glass used for printing of the PUF and a test substrate. The purpose of this test vehicle is to characterise the electrical interconnection without the influence of the ITO coating. Moreover, it also allows mechanical characterisation of the interconnection by shear testing that would not be possible with the fragile ITO-coated glass.

The test substrate has 14 pairs of Cu-pads for test chip mounting (1 mm × 1 mm) and 14 pairs of larger Cu-pads (2 mm × 7 mm) for four point resistance measurement. The length of the copper trace between two pads is 3 mm. The test chip to be mounted on the test substrates has 14 pairs of small Cu-pads (1 mm × 1 mm) connected by a Cu-trace (8 mm). The pitch between pads is 3 mm.

#### 2.1.3. Handling Platform

The handling platform MIMOSE is based on a four-axis Cartesian handling system by LPKF Motion Systems, specifically equipped with gripping, clamping, joining modules depending on the assembly task. The xy-stage carrying a vacuum chuck moves under a granite gantry to which the z-/rotation axis unit is mounted. The control software of the MIMOSE system is programmed in C/C++ by means of an API provided by LPKF Motion Systems. A vacuum gripper, a cartridge dispenser needle unit and a top camera are mounted to the z-/rotation axis unit. The top camera is a Basler acA1920-25uc with a resolution of 1920 px × 1080 px and a variable field of view. Furthermore, there is a bottom camera Basler acA 1300-200uc in combination with a TZ3-2X-32-MA objective and a right-angle adapter with a resolution of 1280 px × 1024 px and a field of view of 3.2 mm × 2.4 mm mounted to the vacuum chuck. The images from these two cameras are acquired by the software Pylon supplied by the camera manufacturer.

### 2.2. Methods

In this section, we introduce the methods for adhesive preparation and in particular for optimisation of the dispensing process. This is followed by methods for mechanical and electrical characterisation of the interconnection.

#### 2.2.1. Conductive Adhesive Preparation

**Figure 2:**
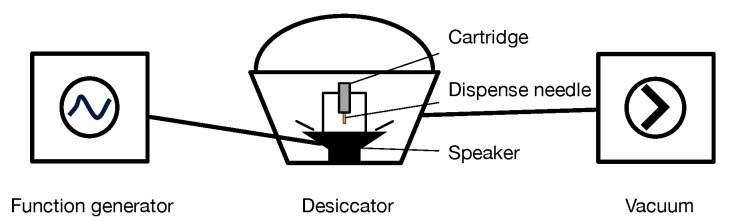
Schematic of the degas-desiccator to remove gas from the adhesive after mixing.

Elecolit 325 is a two-component conductive adhesive. Part A (0.5 g) and part B (0.5 g) are mixed in a 1:1 weight ratio. The mixture is degassed in a desiccator for 20 min. To accelerate degassing, the cartridge is additionally agitated by a speaker (Figure 2). The small amount of adhesive of 1 g used in the present application has a pot time of ≈90 min.

#### 2.2.2. Optimisation of Conductive Adhesive Dispensing

Reproducibility of dispensing processes is influenced by many factors. There are the properties of the material to be dispensed, e.g., insufficient degassing, demixing of multiphase materials, change of material properties over time. Furthermore, dispensing volume tolerance depends on the tolerances of the dispenser controller and on the transfer of the dispensed volume from the dispensing needle to the substrate. In case of pressure time dispensers, the volume pushed out of the dispensing needle depends on the tolerances of the dispenser controller for setting dispensing pressure and dispensing time. For the dispenser Nordson Ultimus I (7017041) used in these test series, the manufacturer specifies a dispensing pressure tolerance of ±2% and a dispensing time tolerance of ±0.5%. These controller tolerances alone already specify an instrument specific lower limit of dispensing reproducibility of ±2.05%. Moreover, transfer of the volume pushed out of the dispensing needle by the pressure pulse onto the substrate is influenced by the wetting properties of the substrate and the needle. Due to time dependent wetting property changes such as residue settling on the needle, not the entire volume may get transferred onto the substrate, but a fraction of it may stick to the needle. This yields one or more small dots followed by a larger dot, when the accumulated volume gets finally transferred from the needle to the substrate. Another influence on dispensing reproducibility are remaining gas bubbles in the adhesive that despite careful degassing after adhesive mixing can not be completely excluded. Previous investigations of pressure time dispensing indicated that dispensing pressure, stand-off distance between substrate and dispenser needle and dispensing time are the three important parameters determining uniform dispensing results for large numbers of adhesive dots. Hence, dispensing pressure is set to 5 bar, the stand-off distance is set to 150 μm and dispensing time is initially set to 500 ms for a target adhesive dot diameter of (1.0±0.2) mm.

**Figure 3:**
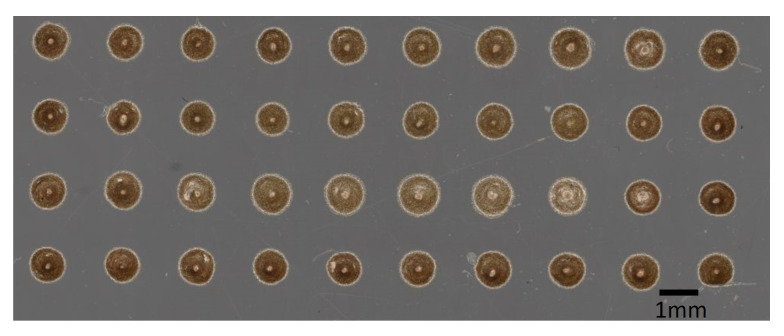
40-dot adhesive dispensing matrix on test foil substrate.

In order to identify dispensing parameters, systematic dispensing tests are conducted by repeatedly dispensing matrices of 40 dots on a foil substrate (40-dot dispensing matrix, Figure 3) and measuring their diameter. In the period between adhesive preparation and the end of pot time 20 matrices can be dispensed.

**Figure 4:**
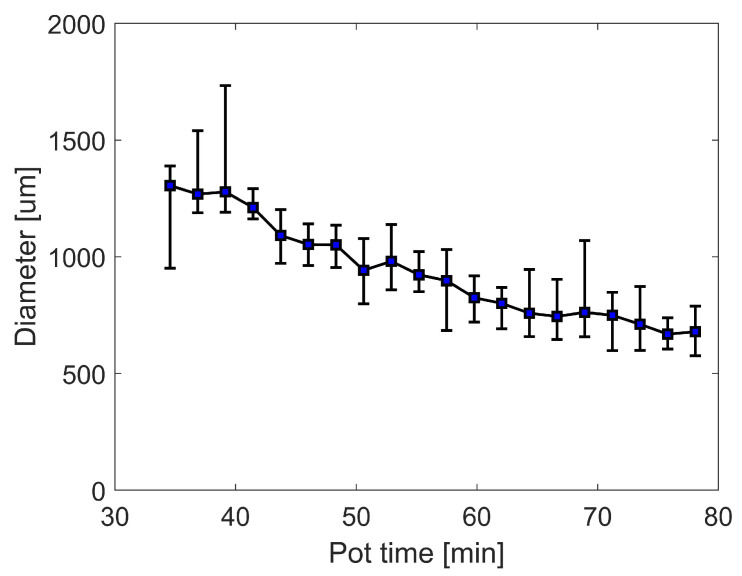
Correlation between pot time of room temperature curing adhesive and diameter of dispensed adhesive dots investigated on test substrates. Average diameter of 40 dots per foil substrate dispensed at different time points (error bars represent min/max values). 20 foil substrates with 40 dots each are consecutively dispensed during 50 min after adhesive preparation (dispensing pressure 5 bar, stand-off distance 150 μm, dispensing time 500 ms).

Dot diameters are measured with a Keyence VHX 7000 microscope. The average diameter of 40 dots on the same foil substrate is shown in Figure 4, error bars represent the maximal and minimal diameters. With all dispensing parameters unchanged, it can be observed that the dot diameters decrease on average from ca. 1300 μm to ca. 600 μm during 50 min after adhesive preparation (Figure 4). This indicates that the adhesive properties change over time already during pot time. We assume that room temperature polymerisation of the adhesive already starts during this period. The resulting increase of viscosity leads to a continuous reduction of the flow rate in the dispensing needle. With fixed dispensing parameters the adhesive dot volume gradually decreases leading to poor reproducibility of the interconnection. Thus, to obtain a series of uniform dots, either dispensing pressure or dispensing time have to be adapted over time.

The measurement of the dot diameter is an insufficient criterion for the dispensed dot volume as dot shape and height information are missing. Thus, the weight of dispensed dots is selected as additional criterion. We derive the dispensing time adaptation Δt for a single dot over time by measuring the mass of the adhesive dots. The density of the conductive adhesive is $\rho =3.2\;g/cm^{3}$. Theoretically, the target mass of a single adhesive dot is $m=1.843\times 10^{-4}\;g$. However, it is difficult to precisely measure such a small mass of a single adhesive dot. Thus, the mass of 40 dots on a foil substrate is measured with a laboratory scale Sartorius ED224s (linearity ±0.0002 g).

**Figure 5:**
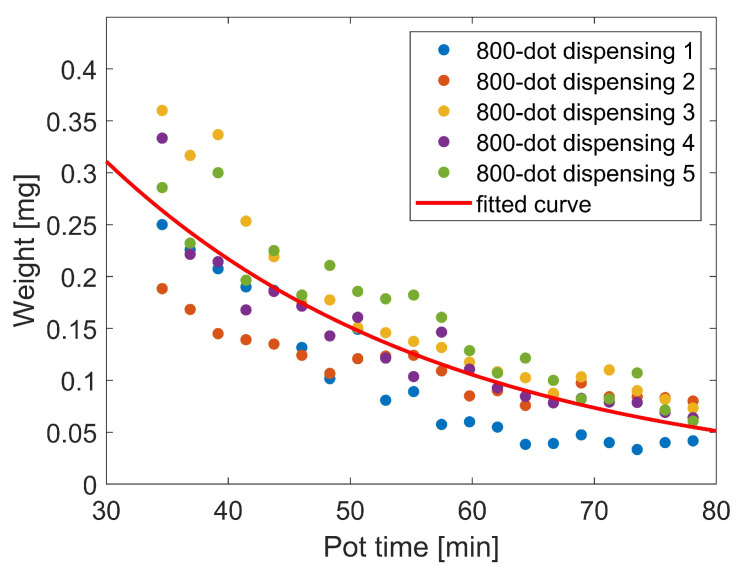
Average mass of an adhesive dot over pot time. Five series of 20 foil substrates with 40 dots each are consecutively dispensed during 50 min after adhesive preparation (dispensing pressure 5 bar, stand-off distance 150 μm, dispensing time 500 ms).

Dispensing pressure is set to 5 bar, the stand-off distance to 150 μm and dispensing time to 500 ms. In total, 800 dots are obtained on 20 foil substrates over 50 min after adhesive preparation. This 800-dot dispensing experiment is conducted five times. For every experiment the adhesive is freshly prepared. After the experiment, every substrate is weighed, the mass of the foil substrate subtracted and the result divided by the number of dots (40) to obtain the average mass of a single adhesive dot. Figure 5 shows the average mass of a single dot dispensed at different time points during the five experiments each covering the period from the end of adhesive preparation to the end of pot time. In contrast to the almost linear decrease of dot diameter these dot mass data show an exponential decrease over time. The Hagen-Poiseuille law (Equation (1)) describes laminar flow through a cylindrical tube. According to this law flow depends on the dispense needle radius (r) to the fourth power, linearly on pressure difference ($\frac{\partial p}{\partial x}$) and inverse linearly on viscosity (η). Laminar flow can be assumed for adhesive dispensing; dispensing pressure has been kept constant in all experiments. Hence, the only remaining factor influencing dispensing volume is a change of viscosity. Lapique has measured the change of viscosity of epoxide based adhesive during curing [33]. He observed an exponential increase of viscosity at the beginning going slowly into saturation for longer curing times. The curve fitted on all collected data points from our five experiments in Figure 5 resembles an inverse of the exponential part at the beginning of such a polymerisation curve. Hence, the assumption of a start of curing of the conductive adhesive already during pot time is confirmed.

(1) $$\begin{array}{c} \dot{V}=\frac{dV}{dt}=-\frac{\pi r^{4}\partial p}{8\eta \partial x} \end{array}$$

Dispensing time is selected as parameter to adapt the dispensing process to the increase of viscosity over time. Based on these considerations, the hypothesis to derive a mathematical relationship between time point *t* and the flow rate Q(t) per millisecond from the measured data is:

(2) $$\begin{array}{c} Q\left(t\right)=\frac{m}{\Delta t}=\frac{ae^{bt+c}}{500} \end{array}$$

With the Matlab data fitting function on all collected data points during the five dispensing runs parameters a, b and c are identified as:

(3) $$\begin{array}{c} a=0.7589,b=-0.0354,c=0.00009 \end{array}$$

The target mass of a single dot is m=0.1843 mg. To obtain a constant weight, with Equation (2), Δt is the dispensing time of a single adhesive dot:

(4) $$\begin{array}{c} \Delta t\left(t\right)=\frac{m}{Q(t)} \end{array}$$

**Figure 6:**
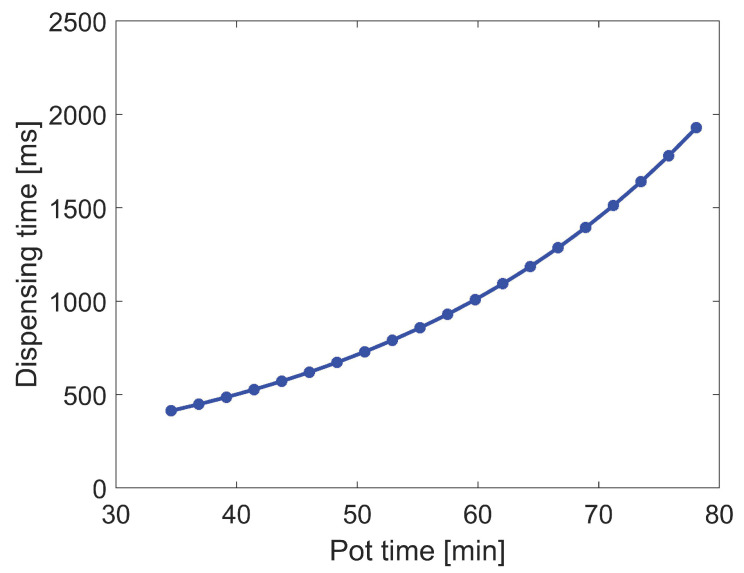
Adapted dispensing time for a single adhesive dot.

Then, the adapted dispensing time of a single adhesive dot Δt over pot time *t* is obtained as shown in Figure 6.

#### 2.2.3. Characterisation

**Figure 7:**
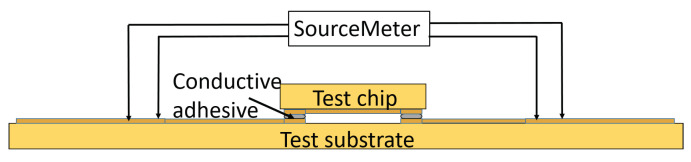
Schematic of the four point resistance measurement for the characterisation of electrical performance of the conductive adhesive interconnection.

Electrical performance of the interconnection is determined by measuring resistance of two adhesive dots connected by Cu-pads and Cu-traces on the test vehicle. Four point resistance measurement (Figure 7) is conducted using a Keithley 2600 Series System SourceMeter and a Formfactor EPS 150 probestation.

**Figure 8:**
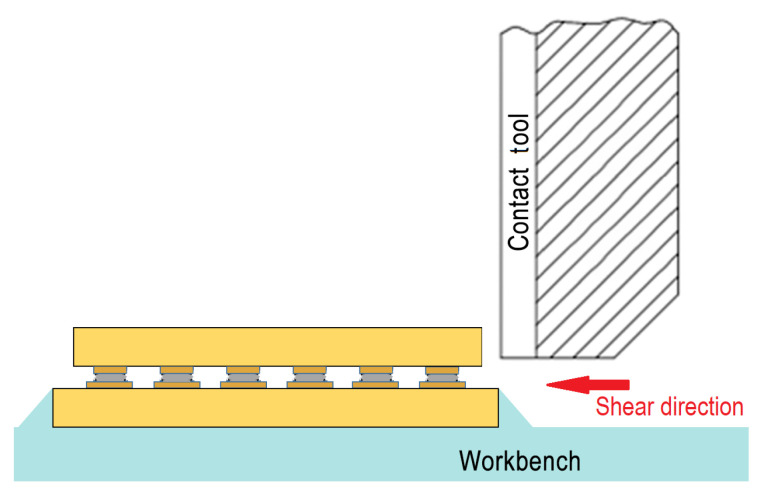
Shear test setup for the characterisation of mechanical stability of the conductive adhesive interconnection.

To assess mechanical stability of the interconnection, a low-speed shear test of the test vehicles is conducted with a Nordson Dage 4000 Multipurpose Bondtester. It conforms to MIL STD 883, where the minimum measuring accuracy of ±5% full scale or 50 g is stated. The tip of shear tester is placed near the chip and parallel to its front side. The shear direction, the relative location of the test-vehicle and the tool are schematically shown in Figure 8.

## 3. Results

The results section is subdivided into the dispensing process improvements achieved and the resulting electrical and mechanical properties of the interconnection. Moreover, the practical application of the optimised dispensing process for automated mounting of circuits printed on ITO-coated glass onto an adapter PCB is outlined.

### 3.1. Reproducibility of the Dispensed Adhesive Volume

**Figure 9:**
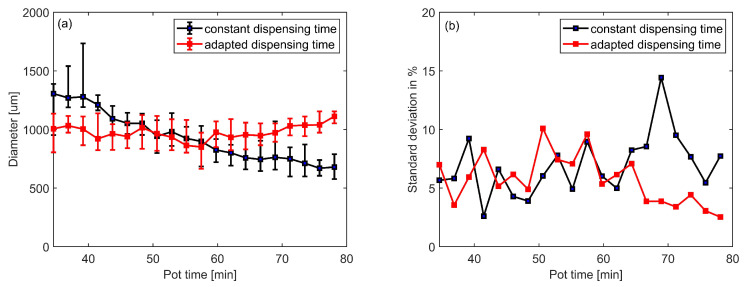
(**a**): Dot diameters over time for constant vs. adapted dispensing time scheme (error bars represent min/max values). (**b**): Standard deviation of dot diameter over time for constant and adapted dispensing scheme.

To verify whether uniformity of adhesive diameter over time is improved with the adapted dispensing time in Figure 6, 800 dots are again continuously dispensed on a foil over 50 min after adhesive preparation. As shown in Figure 9a, the red line indicates the diameters of the adhesive dots dispensed with the adapted dispensing time, the black line with the constant dispensing time of 500 ms. Error bars represent the maximal and minimal diameter of every 40 dots. With the adapted dispensing time, dispensing precision over time significantly improves. Dot diameters vary between ca. 800 μm and 1100 μm. The standard deviation of the diameter is shown in Figure 9b. Before adaptation, the standard deviations of diameter of every 40 dots are between 2.6% and 14.4%. The standard deviation of 800 dots dispensed over 50 minutes after adhesive preparation is 23.0%, while after adaptation, the standard deviations of diameter of every 40 dots are between 2.5% and 10%, the standard deviation of 800 dots is 8.48%, which shows an improvement of dispensing reproducibility. In the later period of pot time, viscosity of the adhesive increases significantly. Under the constant dispensing time scheme, the dispensed volume reduces, however, the stand-off distance remains unchanged at 150 μm. For the reduced volume, the stand-off distance may be no longer appropriate and thus lead to the situation that not the entire volume gets transferred onto the substrate, but a fraction of it sticks to the needle. This would lead to one or more small dots followed by a larger dot, when the accumulated volume gets finally transferred from the needle to the substrate. This is a possible explanation for the higher standard deviation with constant dispensing time in the later period of pot time (around 50 min to 80 min) and the significant improvement with adapted dispensing time in Figure 9a.

### 3.2. Electrical and Mechanical Performance of the Interconnection

**Figure 10:**
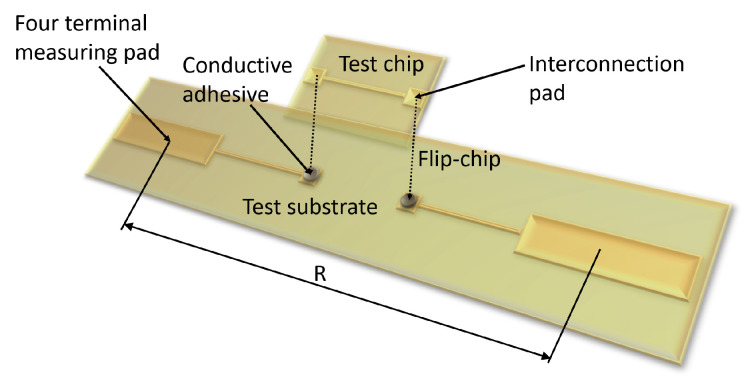
One connection of test vehicle flip-chip mounting.

**Figure 11:**
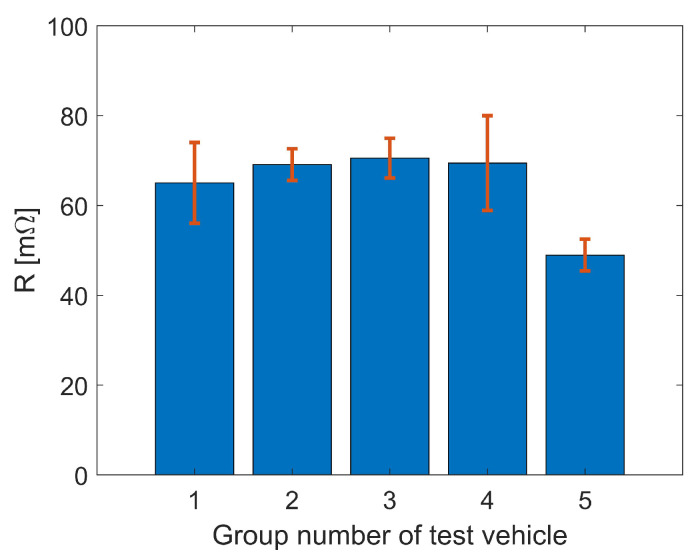
Average resistance of the conductive adhesive interconnection per test vehicle (error bars represent standard deviations).

Electrical performance of the conductive adhesive interconnection is investigated by measuring the resistance of a pair of conductive adhesive dots with the test vehicle. Conductive adhesive is dispensed on the Cu-pads of the test substrate, afterwards, the test chip is placed on the substrate like a flip-chip (Figure 10). Fourteen sets of two conductive adhesive dots connected by Cu-pad and Cu-trace on the test chip are on the same test substrate. They are considered as a group for the measurement. Five groups are assembled and cured at room temperature overnight. The resistance of the Cu-trace on the test vehicle substrate and the test chip is negligible compared to the resistance of the adhesive dots. Resistance of an interconnection is measured with four point measurement. Figure 11 shows the resistance measurement results. When calculating the resistances of all 70 pairs on five groups, the average resistance is 64.54 mΩ with a standard deviation of 10.49 mΩ (16.2%).

**Figure 12:**
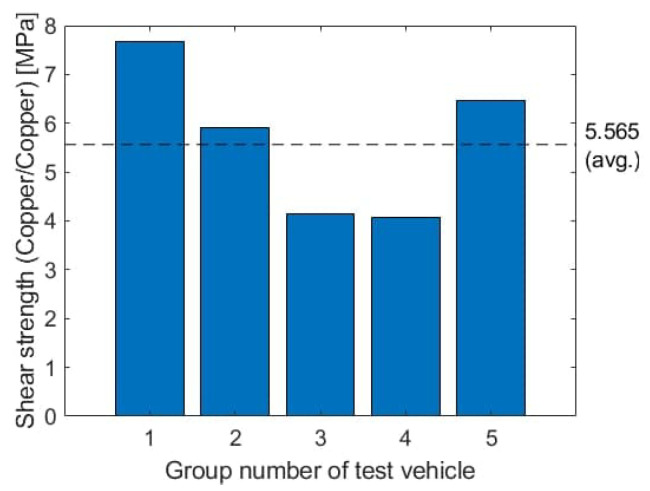
Shear strength per interconnection.

**Figure 13:**
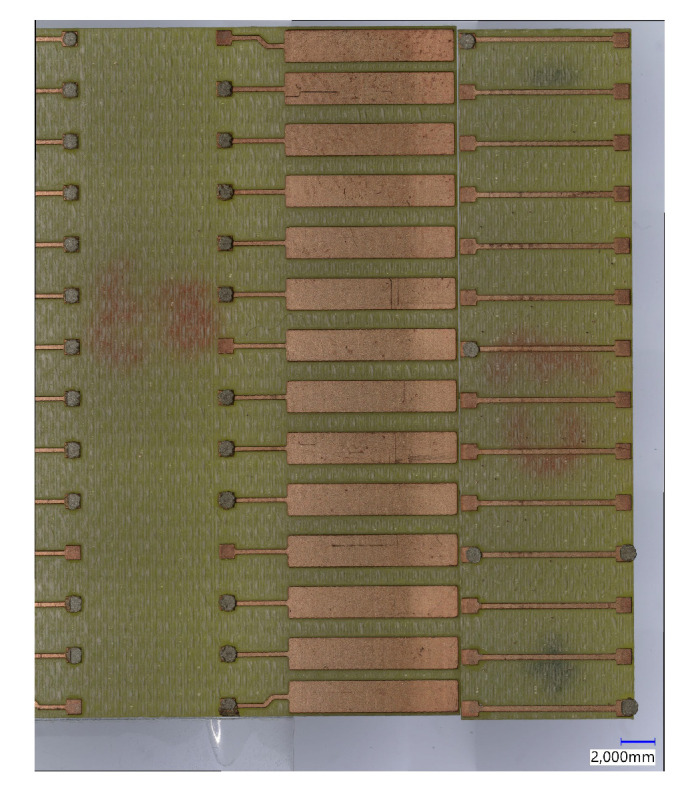
After the shear test: conductive adhesive is observed remaining completely either on the Cu-Pad of test chip or on the Cu-pad of the test substrate.

Shear strength values are shown in Figure 12. The average shear strength for the interface copper pad-conductive adhesive-copper pad of five test vehicles is 5.65 MPa. The lap shear strength value (steel-conductive adhesive-steel) provided by the adhesive manufacturer is 5 MPa to 10 MPa [34]. Three out of five samples lie in this nominal range. Visual inspection of the samples after shear testing shows that the interconnection frequently fails on the interface between conductive adhesive dot and copper pad and not in the bulk of the adhesive (Figure 13). This is a possible reason that the full shear strength of the adhesive is not in any case achieved.

### 3.3. Automated Mounting Process for Hybrid Printed Systems

In this work, we develop an automated mounting process for integrating hybrid components onto solid or flexible substrates. This mounting process derived from flip-chip-technology has been utilized for integrating printed circuits on an ITO-coated glass onto an adapter PCB as a case study.

#### 3.3.1. Standardised Interface for Interconnecting Circuits Printed on ITO-Coated Glass to PCB

In this section, the mounting process of an ITO-coated glass (20 mm × 20 mm) with printed electronic devices onto an adapter PCB is shown. Firstly, a standardised layout of the interface is defined. As a result, the standard layout contains a free area in its center available for the application specific printed circuit and predefined pads around its e.g., as interconnection interface to an adapter PCB. In a first version, the interface consists of seven ITO-pads at every edge of the ITO-coated glass. Two fiducial marks are positioned in two diagonal corners for orientional and positional alignment with high precision. The position and structure of the ITO-pads for interconnection to the adapter PCB can be tailored to the application. For the sake of process and interconnection robustness, in a first version the dimensions of the ITO-pads as well as the contact pads on the adapter PCB are 1 mm × 3 mm and 1 mm × 1 mm, respectively. The spacing between two pads is 1 mm. The adapter PCB has 36 gold-coated contact pads, 28 of which mate with contact pads on the ITO-coated glass to realise electrical and mechanical interconnection between the bond partners. The remaining eight pads are currently not used in the first application for the PUF.

**Figure 14:**
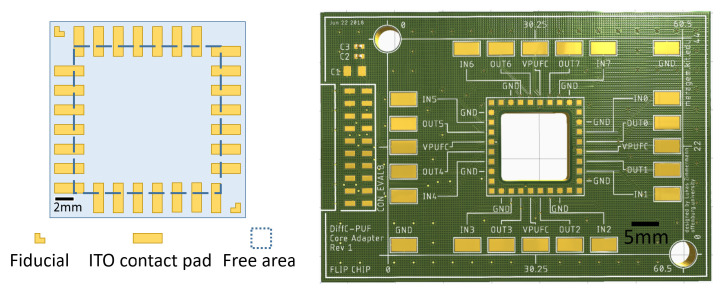
Schematic drawing of the ITO-coated glass and adapter PCB.

The ITO-coated glass (20 mm × 20 mm) and the adapter PCB are shown in Figure 14. The ITO layer is structured by laser processing to fabricate the wiring for the circuits to be printed and the contact pads for the interface to the adapte PCB.

#### 3.3.2. Automated Mounting Process of Circuits Printed on ITO-Coated Glass to PCB

**Figure 15:**
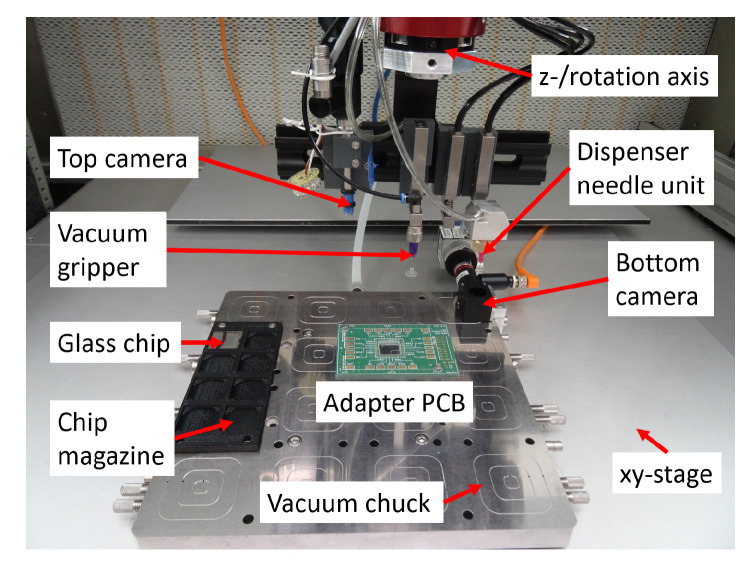
Handling platform MIMOSE equipped with ITO-coated glass chip and adapter PCB for the automated mounting process.

To realise the automated mounting process, the four axis handling platform MIMOSE is applied (Figure 15). The ITO-coated glasses are supplied in a part specific glass supply magazine and face down suspended on a frame to protect the printed structures in the central area. The adapter PCB is clamped against reference pins on the vacuum chuck. The position of the adapter PCB is acquired with the top camera. The bottom camera system is used for identification of the fiducials on the ITO-coated glass from below.

**Figure 16:**
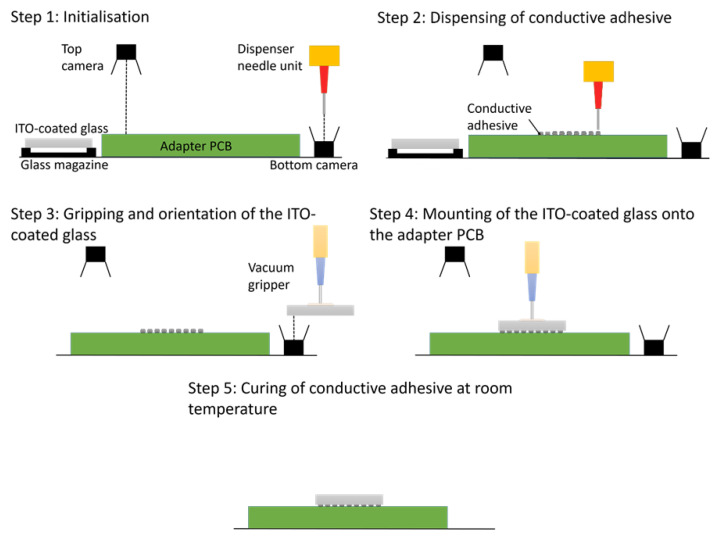
Process for automatic mounting of ITO-coated glass onto an adapter PCB with a four-axis handling system using image processing, adapted adhesive dispensing and vacuum gripping.

Prior to the mounting process, the ITO-coated glass is stored in the glass supply magazine, one adapter PCB is placed manually in the handling platform, and the mixed and degassed conductive adhesive is filled into the dispenser needle unit. Starting with the initialisation of the machine, the adapter PCB and the dispenser needle are automatically referenced by an initialisation program based on an in-house image processing routine. Afterwards, a pre-dispensing step is executed to clean the dispensing needle; a precondition for reliable and reproducible adhesive flow. Subsequently, the adhesive is automatically dispensed onto the adapter PCB contact pads. The ITO-coated glass is then automatically gripped by a vacuum gripper. Images of the fiducials on the ITO-coated glass are acquired by the bottom camera and offsets are calculated by the image processing software for precise positioning and orientation of the ITO-coated glass over the target position on the adapter PCB. By respective motions of the x, y, z- and rotation axes, the ITO-coated glass is then settled on the dispensed conductive adhesive dots. After shutting off the gripper vacuum, the machine moves back to the home position. Finally, the assembled device is manually removed and the next adapter PCB is placed on the platform. The mounting process is then repeated with the next ITO-coated glass. Final mechanical stability and electrical conductivity of the interconnection are achieved after curing the assembly at room temperature for more than 16 h. The complete automated mounting process workflow is shown in Figure 16.

#### 3.3.3. Case Study Physically Unclonable Function

In the use case outlined in this paper, the above process is applied to realise a novel printed hardware-security device in the form of a physically unclonable function (PUF). A PUF describes a hardware-security primitive, which allows for fingerprinting. Here, intrinsic variation in printed electronics is exploited to generate unique identifiers. The intrinsic variation source, which is labeled as PUF core, consists of the inkjet-printed inverter array, which is fabricated in the central area of the ITO-coated glass. The input-/output ports of the inverter array are connected to the standard interconnection interface on the edges of the ITO-coated glass. The inverter array is based on low-voltage electrolyte-gated transistors (EGT), whose channel is realised by inkjet-printing of an indium (III) nitrate hydrate (H2InN3O10)-based precursor. For the subsequent formation of the indium-oxide (In2O3) thin-film semiconductor channel, an annealing step at 400 °C is applied. ITO-coated glass both fulfils the high temperature requirement resulting from the annealing step and allows creation of wiring, resistive meander structures, EGT terminals and input-/output ports by laser structuring. The gate insulator and the top-gate electrode are inkjet-printed using a composite solid-polymer-electrolyte (CSPE) and poly(3,4-ethylenedioxythiophene):poly (styrenesulfonate) (PEDOT:PSS), respectively. The deployed circuit architecture and devices can be found in [23].

**Figure 17:**
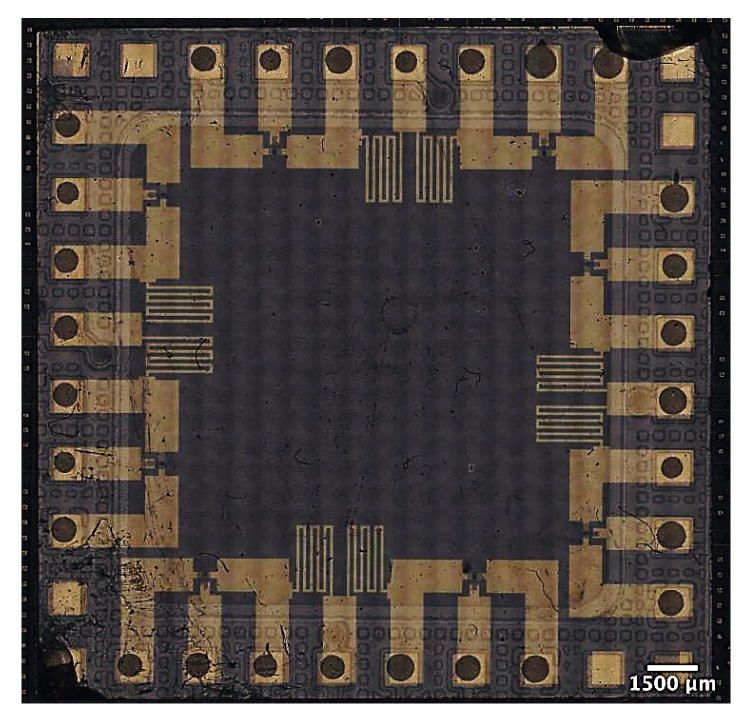
Mounted ITO-coated glass with printed EGTs.

The developed mounting process enables seamless integration of the printed PUF cores on ITO-coated glass onto a specially designed adapter PCB (Figure 17). The adapter PCB is attachable to the PUF’s control logic and signal processing layer, which consists of classical silicon-based electronics. Merging both technologies in novel ways enables new applications in the field of hybrid printed systems. The design, fabrication workflow, and the corresponding results of the printed PUF circuit on the ITO-coated glass are shown in [23]. The developed automated integration approach enables fabrication of larger batch sizes of PUF instances, which allows for PUF metric investigation. Due to the developed room-temperature mounting process, any potential adverse influences from temperature on these complex printed active devices based on different novel material inks can be excluded, before commissioning of the printed system.

## 4. Conclusions

In this paper, we have introduced a novel automated room temperature bumpless flip-chip mounting process for hybrid printed electronics based on isotropic conductive adhesive. Firstly, the dispensing process of the room temperature curing adhesive Panacol Elecolit 325 was optimised in order to compensate for increasing adhesive viscosity that occurs already during pot time. By implementing a function to correct these time dependent viscosity changes, the reproducibility of dispensed adhesive dots and thus of the electrical interconnections is guaranteed over the entire processing time. PCB test vehicles were used to perform four point resistance measurements and shear tests to characterize the electrical and mechanical performance of the interconnection. Further, a standardised interconnection interface has been defined between ITO-coated glasses containing inkjet-printed structures and an adapter PCB with peripheral electronic components. The developed mounting process and interconnection interface were applied to automatically mount novel printed security devices to adapter PCBs. A printed security device consists of four PUF cores in the centre of the ITO-coated glass.

The proposed automated mounting process enables reproducible fabrication of hybrid printed devices based on ITO-coated glass in larger batch sizes than the manual assembly process used beforehand. Thus, more comprehensive evaluation of the device parameters is now possible. The standardized interconnection interface allows for straightforward transfer of the process to any other circuit printed on ITO-coated glass. Due to the room temperature processing, any thermal influence of the mounting process on the performance of the complex printed circuits can be excluded. The herein described dispensing time correction methodology can be adapted to different room temperature curing adhesives and/or different dot diameters. The optimised dispensing process and the design of the standard interconnection both hold potential for further miniaturisation and thus, higher integration density. First tests have shown that with the same adhesive, dots down to 200 μm in diameter can be reproducibly dispensed.

Thus, the developed automated room temperature bumpless flip-chip mounting process is not limited to the interconnection of devices printed on ITO-coated glass to adapter PCBs. It is a promising approach for future utilization in numerous hybrid printed electronic circuits that are based on novel and often temperature sensitive materials both on the side of substrates and inks. Furthermore, it can be applied to enable novel hybrid systems by combining the functionality of standard electronic components with emerging research and application fields in flexible electronics, e.g., [35,36].

## Data Availability

The data that support the findings of this study are available from the corresponding author upon reasonable request.

## Abbreviations

The following abbreviations are used in this manuscript:

ITO	Indium tin oxide
FHE	Flexible hybrid electronics
EGT	Electrolyte-gated field effect transistors
PEDOT:PSS	Poly(3,4ethylenedioxythiophene):poly(styrenesulfonate)
PCB	Printed circuit board
PUF	Physically unclonable function
